# Evaluation of a Novel Thermosensitive Heparin-Poloxamer Hydrogel for Improving Vascular Anastomosis Quality and Safety in a Rabbit Model

**DOI:** 10.1371/journal.pone.0073178

**Published:** 2013-08-27

**Authors:** Ying-Zheng Zhao, Hai-Feng Lv, Cui-Tao Lu, Li-Juan Chen, Min Lin, Ming Zhang, Xi Jiang, Xiao-Tong Shen, Rong-Rong Jin, Jun Cai, Xin-Qiao Tian, Ho Lun Wong

**Affiliations:** 1 Wenzhou Medical University, Wenzhou City, Zhejiang Province, China; 2 College of Medicine, Drexel University, Philadelphia, Pennsylvania, United States of America; 3 Departments of Pediatrics and Anatomical Sciences and Neurobiology, University of Louisville School of Medicine, Louisville, Kentucky, United States of America; 4 Department of Ultrasonography, the Second Affiliated Hospital of Wenzhou Medical University, Wenzhou, China; 5 School of Pharmacy, Temple University, Philadelphia, Pennsylvania, United States of America; Aristotle University of Thessaloniki, Greece

## Abstract

Despite progress in the design of advanced surgical techniques, stenosis recurs in a large percentage of vascular anastomosis. In this study, a novel heparin-poloxamer (HP) hydrogel was designed and its effects for improving the quality and safety of vascular anastomosis were studied. HP copolymer was synthesized and its structure was confirmed by Fourier transform infrared spectroscopy (FTIR) and nuclear magnetic resonance spectroscopy (^1^H-NMR). Hydrogels containing HP were prepared and their important characteristics related to the application in vascular anastomosis including gelation temperature, rheological behaviour and micromorphology were measured. Vascular anastomosis were performed on the right common carotid arteries of rabbits, and the *in vivo* efficiency and safety of HP hydrogel to achieve vascular anastomosis was verified and compared with Poloxamer 407 hydrogel and the conventional hand-sewn method using Doppler ultrasound, CT angiograms, scanning electron microscopy (SEM) and histological technique. Our results showed that HP copolymer displayed special gel-sol-gel phase transition behavior with increasing temperature from 5 to 60 °C. HP hydrogel prepared from 18 wt% HP solution had a porous sponge-like structure, with gelation temperature at approximately 38 °C and maximum elastic modulus at 10,000 Pa. In animal studies, imaging and histological examination of rabbit common jugular artery confirmed that HP hydrogel group had similar equivalent patency, flow and burst strength as Poloxamer 407 group. Moreover, HP hydrogel was superior to poloxamer 407 hydrogel and hand-sewn method for restoring the functions and epithelial structure of the broken vessel junctions after operation. By combining the advantages of heparin and poloxamer 407, HP hydrogel holds high promise for improving vascular anastomosis quality and safety.

## Introduction

Vascular anastomosis is the cornerstone of vascular, cardiovascular and transplant surgery [1]. In 1912, Alexis Carrel was awarded the Nobel Prize in Physiology and Medicine due to his contribution in improving vascular closure technology [2]. During the following 100 years, various new techniques developed, including medical adhesive anastomosis, cuff vascular anastomosis, built-in soluble stent method and laser welding method [3–8]. However, restenosis and thrombosis remain significant limitations to the development of vascular anastomosis, especially for small blood vessels [9–13]. Causing necessity for further intervention, restenosis not only has clinical but also economic implications.

Heparin, including low molecular weight heparin (LMWH) and other types of heparin, is one of the most intensively studied glycosaminoglycans because of its anticoagulant properties [14]. LMWH is a biocompatible, biodegradable and water-soluble natural polysaccharide. According to the response-to-injury hypothesis [15], restenosis is caused by intimal hyperplasia due to proliferation and migration of vascular smooth muscle cells (SMC) from the media into the intima. LMWH may inhibit cell proliferation and intimal hyperplasia [16].

Poloxamer block copolymers have been introduced in the late 1950s and since then they have been proposed for diverse pharmaceutical applications [17,18]. This group of copolymers consists of ethylene oxide (EO) and propylene oxide (PO) blocks arranged in a triblock structure EO_x_-PO_y_-EO_x_. Registered trademarks of these copolymers (e.g., Pluronic, Synperonic or Tetronic) cover a large range of liquids, pastes and solids. They are synthesised by sequential polymerisation of PO and EO monomers in the presence of sodium hydroxide or potassium hydroxide [19]. Poloxamer 407, a nontoxic poly(ethylene oxide)/poly(propylene oxide)/poly(ethylene oxide) (PEO/PPO/PEO) triblock copolymer with a weight-average molecular weight of 12,600, contains 70% hydrophilic ethylene oxide units and 30% hydrophobic propylene oxide units. FDA guide has presented Poloxamer 407 as an inactive ingredient for different types of preparations (e.g., IV, inhalation, oral solution, suspension, ophthalmic or topical formulations). Aqueous solutions of PEO–PPO–PEO block copolymers exhibit temperature induced aggregation phenomena as a result of the hydrophobic nature of the PPO block. An aqueous solution of poloxamer 407 at 20% or higher concentration transforms from a viscous solution to a semi-solid gel by increasing the temperature from 4 °C to body temperature (37 °C), and this gelation is reversible by decreasing the temperature [20,21]. With the thermosensitive character, poloxamer 407 was used as a filling agent in a new method of sutureless and atraumatic vascular anastomosis--medical adhesive anastomosis [1]. This new technology showed potential for improving efficiency and outcomes in the surgical treatment of cardiovascular disease.

Combining the advantages of LMWH and poloxamer 407, a novel thermosensitive material--heparin-poloxamer (HP) was synthesized in our laboratory. From the characterization observation, HP conjugate shows about 60% anticoagulant property as LMWH, while maintaining similar phase-change performance as poloxamer 407. As an upgrade substitute of poloxamer 407, HP hydrogel may play the dual roles of thermosensitive filling agent and anti-restenosis agent in vascular anastomosis.

In this study, HP hydrogel was prepared and experimented to improve the vascular anastomosis quality and safety. The structure of HP was confirmed by Fourier transform infrared spectroscopy (FTIR) and nuclear magnetic resonance spectroscopy (^1^H-NMR). Gelation temperature, rheological behaviour and micromorphology of HP hydrogel were measured to evaluate its important characteristics related to the application in vascular anastomosis. In experiment *in vivo*, the right common carotid arteries of rabbits were used to perform vascular anastomosis. Doppler ultrasound, CT angiograms, scanning electron microscopy (SEM) and histological examination were applied to verify the efficiency and safety of HP hydrogel in vascular anastomosis. Compared with hand-sewn group (traditional sutured anastomoses) and poloxamer 407 group, the advantages of HP hydrogel in vascular anastomoses were discussed.

## Materials and Methods

### 1: Materials

Poloxamer 407 was purchased from BASF (Shanghai, China). Triethylamine, 4-nitrophenyl chloroformate, 4-dimethylaminopyridine, 1-ethyl-3-(3-dimethylaminopropyl)-carbodiimide (EDC), and N-hydroxysuccinimide (NHS) were purchased from Aldrich Chemical Company (Shanghai, China). Low molecular weight heparin sodium salt (from porcine intestinal mucosa, 140 unit/mg) was supplied by Freda Biochem Company (Jinan, China). 2-octylcyanoacrylate was purchased from Beijing ShumKong Medical Company (Beijing, China). All other chemicals were used as received without further purification.

Japanese white rabbits (2-2.5 kg) were housed in an approved animal care center and were provided with standard chow and water *ad libitum*. The laboratory animal ethics committee and laboratory animal centre of Wenzhou Medical University approved all rabbit studies.

### 2: Preparation of HP copolymer

#### Synthesis of mono amine-terminated Poloxamer (MATP)

**Figure 1:**
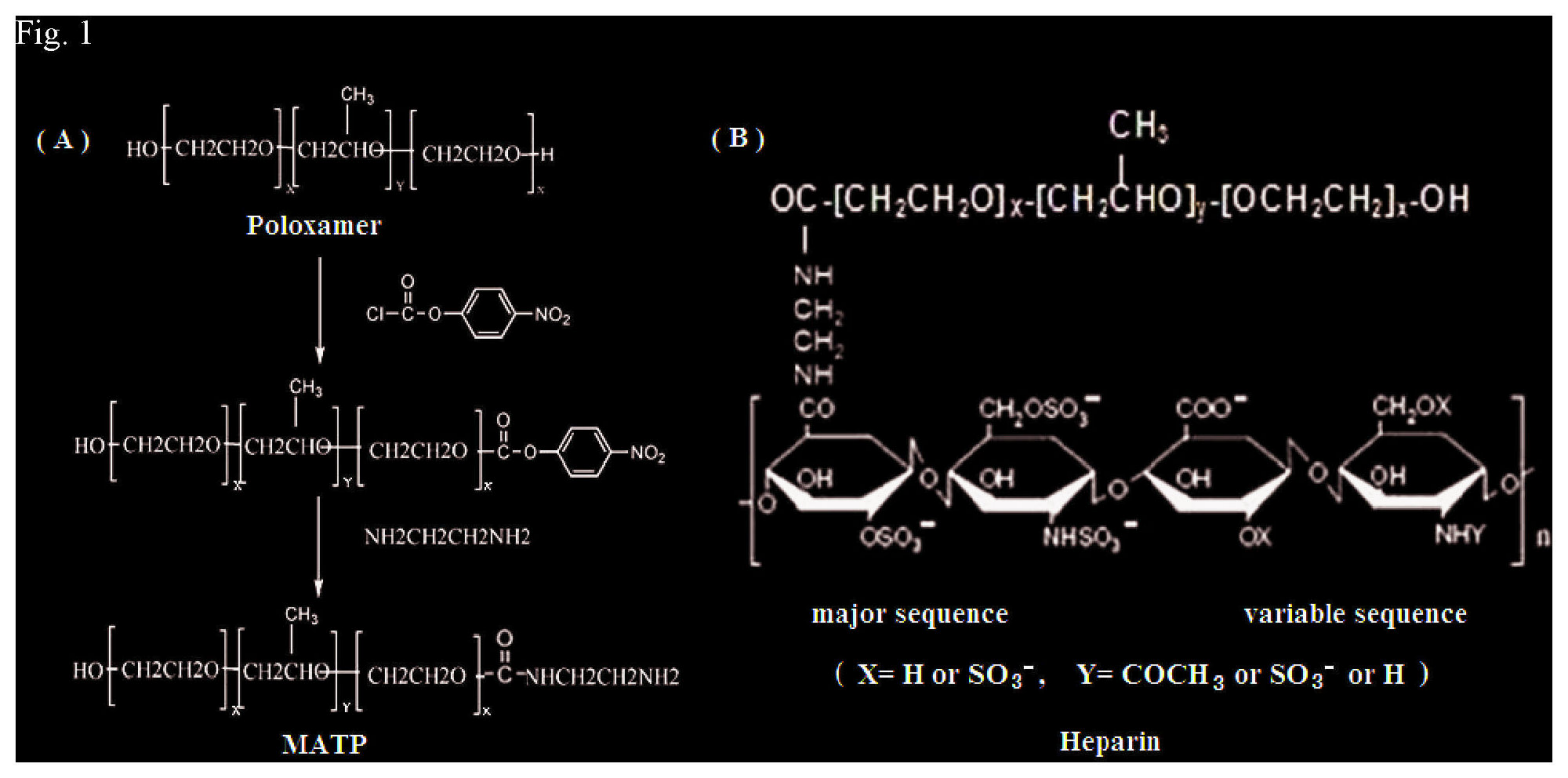
Synthetic route and chemical structure of heparin-poloxamer (HP) conjugate. (A: Synthetic route of amine-terminated poloxamer 407; B: Chemical structure of HP conjugate).

The Synthesis of MATP followed an established protocol (Figure 1A). MATP was prepared by two-step reactions [22]. In the first step, 1 mM poloxamer 407 reacted with 1.3 mM 4-nitrophenyl chloroformate dissolved in 20 ml methylene chloride in the presence of triethylamine at room temperature for 4 h to yield a 4-nitrophenyl formate-derivatized intermediate. Using petroleum ether as extract solution, the intermediate was recovered by extraction for three times. In the second step, 1mM intermediate reacted with 3mM diaminoethylene dissolved in 20 ml methylene chloride at room temperature overnight. After reaction, the mixture was extracted three times with petroleum ether and then dialyzed against distilled water using a membrane with a molecular weight cutoff of 3500 for 3 days at room temperature, and finally lyophilized to obtain the product.

#### Synthesis of HP

HP was prepared according to EDC/NHS method. MATP (4.5 g, 0.5 mM) was coupled with low molecular weight heparin salt (2.5 g, 0.5 mM) by EDC (0.106 g, 0.5 mM) and NHS (0.032 g, 0.25 mM) in MES buffer (0.5M, pH 5.6) for 24 h at room temperature. Specifically, reaction between the amine groups of poloxamer 407 and carboxyl ones of heparin in the presence of EDC resulted in amide bond formation. After the reaction the mixture was dialyzed against distilled water using a dialysis bag (MWCO = 14000) for 3 days and then lyophilized to obtain the product (Figure 1B).

### 3: Characterization of HP copolymer

#### 3.1: FTIR and ^1^H-NMR

The structure of HP copolymer was characterized using Fourier transform infrared spectroscopy (FTIR) (670 FT-IR, Nicolet, Madison, WI, USA). Hydrogen-1 nuclear magnetic resonance spectroscopy (^1^H-NMR) (AVANCE III 600 MHz, Bruker, Fallanden, Switzerland) measurement was carried out to confirm the change of chemical structure of HP.

#### 3.2: Anti-coagulant activity of HP copolymer

The recalcification time test was used to reflect the anti-coagulant activity of HP copolymer. Three HP concentrations: 2.9 mg/ml (high level), 5.8 mg/ml (medium level), 9.5 mg/ml (low level) were tested. HP solution was added into a test tube containing 0.2 mL human plasma under 37 °C water bath, followed by the addition of 0.2 mL plasma and 0.2 mL CaCl_2_ solution (0.025M). When filament substance initially appeared in the tube, the time was recorded as the index for recalcification. For comparison, 0.5 mg/ml LMWH solution was tested using the same procedure.

### 4: Preparation of HP-based hydrogels and Poloxamer hydrogels

HP-based hydrogels containing different amounts of HP were prepared using the cold method [23,24]. In brief, lyophilized HP powder was mixed in PBS at 4 °C with gentle stirring. The mixture was kept in a refrigerator at 4 ^o^C overnight until a clear solution was formed. Table 1 shows the chemical compositions of HP hydrogels prepared.

**Table 1:** Compositions of the HP hydrogels.

*Group*	*HP concentration (wt%)*						
	15.5	16	17	18	19	20	22
HP (g)	0.775	0.800	0.850	0.900	0.950	1.000	1.100
PBS (g)	4.225	4.200	4.150	4.100	4.050	4.000	3.900

For the purpose of comparison, we also prepared poloxamer 407 hydrogel using the same procedure.

### 5: Gelation temperature measurement

A 15-ml transparent vial containing 5 ml of aqueous poloxamer 407 or HP copolymer solution at different concentrations (set as 2.4 section mentioned) was placed in a water bath and heated at a rate of 0.5°C min^-1^ (from 22 ^o^C up to 60 ^o^C) Gel formation was indicated by a lack of movement of the meniscus on tilting the vial; the temperature at which immobility of the meniscus in each vial was first noted was recorded as the sol–gel transition temperature at that concentration. The transition temperature was measured in triplicate and the average value of each point was reported [25].

### 6: Rheological behavior of HP hydrogel

Rheological measurements of the HP hydrogels were carried out at different temperatures using a DYNALYZER2000 (Rheological, Sweden) depending on their concentration (16 and 20 wt. %). The samples were prepared in a PBS buffer (pH = 7.2-7.4). The elastic (G') and viscous (G″) shear moduli were measured at a frequency of 0.1 Hz. A parallel plate geometry was used (plate diameter = 25 mm, gap = 0.45 µm and stress = 10 Pa) for the oscillatory shear rheological measurements. The hydrogel sample was placed on the plate of the rheometer and the temperature was increased at 2°C intervals over the range 5-60°C [26].

### 7: Micromorphology of HP hydrogel

The micromorphology of the dehydrated HP hydrogel at 18 wt% was observed by SEM. The hydrogels were immersed into liquid nitrogen and freeze-dried at -50 °C. For SEM analysis, the HP hydrogel specimens were freeze-dried under vacuum for 1 day. The dehydrated specimens were cross-sectioned and sputter-coated with gold, and their surface morphology was observed in a scanning electron microscope (Hitachi, H-7500, Japan) [27,28].

### 8: Thermoreversible HP hydrogel and cyanoacrylate glue sutureless anastomoses in vivo

#### 8.1: Animals

Twenty-four Japanese White Rabbits (2–2.5 kg) were used in this study. Consent and approval for this investigation were obtained from the Laboratory animal Ethics Committee of Wenzhou Medical University (Wenzhou, Zhejiang, China). All animal experiments are in accordance with the recommendations of the Laboratory animal Ethics Committee of Wenzhou Medical University. The rabbits were acclimatized on site for at least 7 days before surgery.

#### 8.2: Operation procedure

All surgical procedures were performed under intravenous anesthesia with 3% pentobarbital sodium 1mL·kg^-1^. The surgical procedures were carried out under sterile conditions. The prepared area was isolated with sterile drapes to ensure sterility throughout the procedure. All anastomoses were carried out at the right common carotid arteries. Using surgery scissors, a midline neck incision was made, and the right jugular artery was exposed by incising the platysma and neck muscles. The vagus nerve was dissected carefully from the common jugular artery to allow manipulation of the artery without inducing bradycardia in the rabbit.

The mean outer diameter of the blood vessels was between 1.88 and 2.30 mm. Two vascular clamps were applied to the carotid artery, which was then transected using microscissors. After flushing the two stumps of the vessel with 0.9% sodium chloride solution, surrounding connective tissue was removed and the two vessel stumps prepared for the anastomotic procedure.

**Figure 2:**
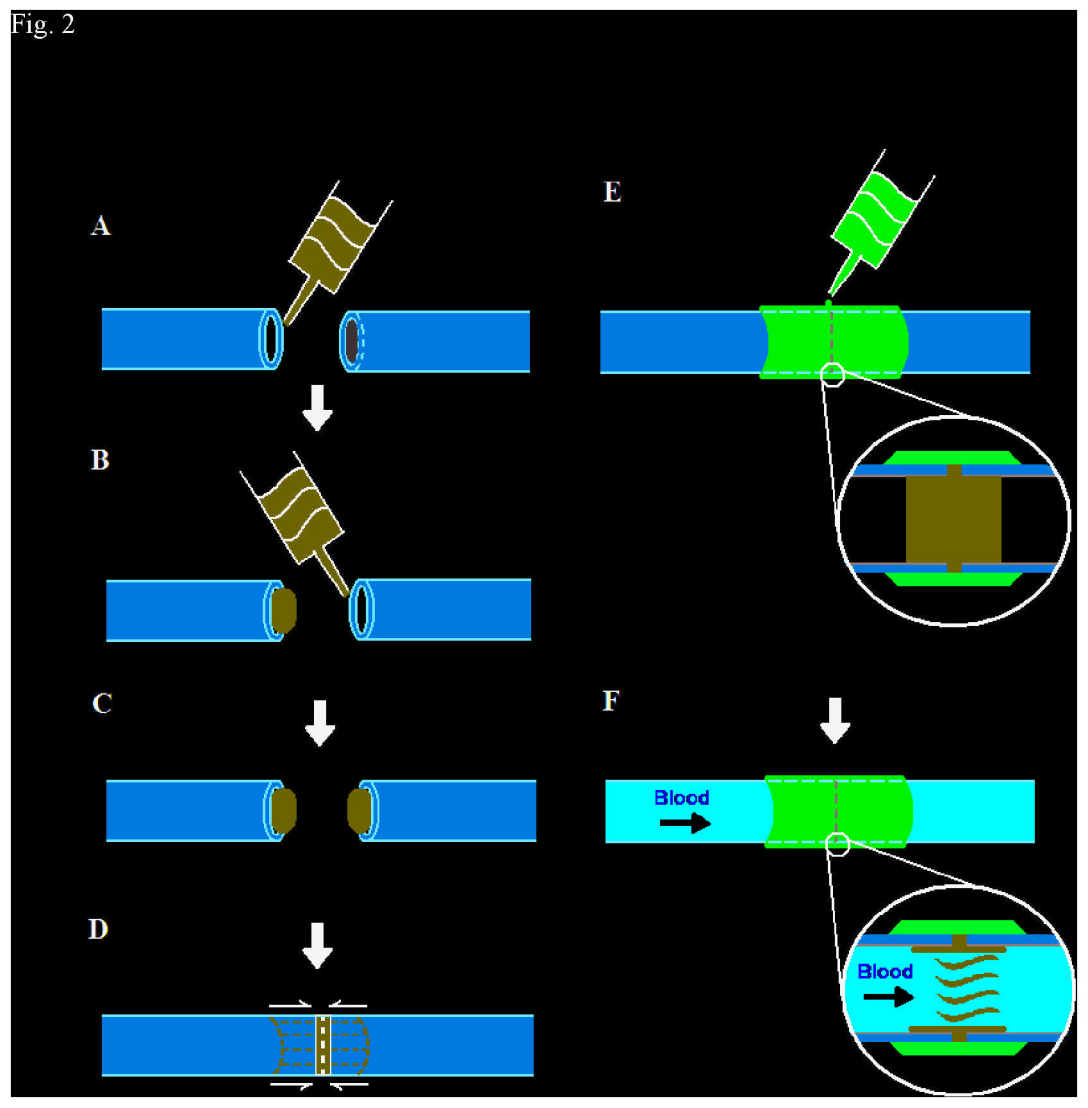
Procedures of medical adhesive anastomosis combined with HP hydrogel. (A: Fractured vessels; B: Liquid HP was introduced into one side of the broken vessel; C: Liquid HP was introduced into the other side of the broken vessel, and transferred into solid phase under controlling environmental temperature; D: Alignment of HP perfused vessels; E: Coating of cyanoacrylate on the outer periphery of the vascular junction; F:HP was transferred from solid to liquid under *in vivo* temperature, and blood flowed through the anastomosis junction).

Medical adhesive anastomosis: The HP hydrogel formulation or poloxamer 407 hydrogel formulation (see Table 1) was heated above the transition temperature and infused it into the divided blood vessel ends to maintain an open lumen. The procedures of medical adhesive anastomosis combined with poloxamer 407 or HP hydrogel were shown in Figure 2. A radiant halogen heat source was used to maintain the surgical field temperature precisely above the phase-transition temperature to achieve the maximum elastic modulus, but below the 43°C threshold where thermal damage occurs. After precise approximation of the intimal layer of the vessel, we applied 2-octylcyanoacrylate solution without dilution in a circumferential manner to complete the anastomosis. On removal of the heat source, the temperature of the operative field quickly returned to ambient temperatures (below the poloxamer transition temperature). Patency was assessed with a milking test immediately following removal of the vascular clamps. Once the anastomosis had been completed, wound muscle layers were sutured closed with 4/0 Vicryl Rapide, and the skin was closed with 2/0 Vicryl Rapide. For all animals the time taken from the moment the vascular was clamped until the clamps were released was noted. After surgery the animals received 0.5 mg/kg amoxicillin and clavulanic acid injected subcutaneously as a bolus antibiotic prophylaxis. Pain management consisted of a single subcutaneous dose of 0.03 mg/kg buprenorphine as motor function returned.

As the traditional sutured anastomoses, hand-sewn operations were performed for comparison. Sham-operated group was also used as the normal control. No intraoperative or postoperative complications were encountered in either group. All rabbits recovered without complication. They remained ambulatory and thriving until the date they were killed.

### 9: Imaging evaluation

Vessel diameter and flow were measured at 6 weeks and 6 months after operation (n = 5 per group) using Doppler ultrasound (Siemens, ACUSON S2000). CT angiograms with intravenous Omnipaque (ART) contrast were performed to assess patency at 6 weeks and 6 months (n = 5 per group). In addition, scanning electron microscopy at 12 months after operation (n = 3 per group) were detected using the FEI Tecnai TF 30 He (Polara) electron microscope equipped with a field emission gun and Tietz 224HD 2K×2K charge-coupled device camera.

### 10: Histological examination

At 12 weeks (n = 5) after operation, the anastomoses was isolated, rinsed with saline solution, and fixed in 4% (vol/vol) paraformaldehyde, embedded in paraffin using an embedding center and sectioned into 5µm slices. The slices were stained with hematoxylin-eosin and observed under a light microscope [29–31].

The percentage increase of the intima thickness at week 12 after operation was calculated with the following equation.

$$\text{Percentage increase of the intima thickness (\%)}=\frac{\text{W'-W}}{\text{W}}\times 100$$

Where W’ was the intima thickness of experimental group at week 12 after operation; W was the intima thickness in normal rabbits.

### 11: Statistical analysis

One-way ANOVA and Student’s t-test or Kruskal-Wallis test were adopted for statistical comparison using the SAS 8.01 (1999-2000, SAS Institute Inc., Cary, NC, USA). The data difference was considered to be statistically significant when the *P*-value was less than 0.05.

## Results and Discussion

### 1: Characterization of HP copolymer

**Figure 3:**
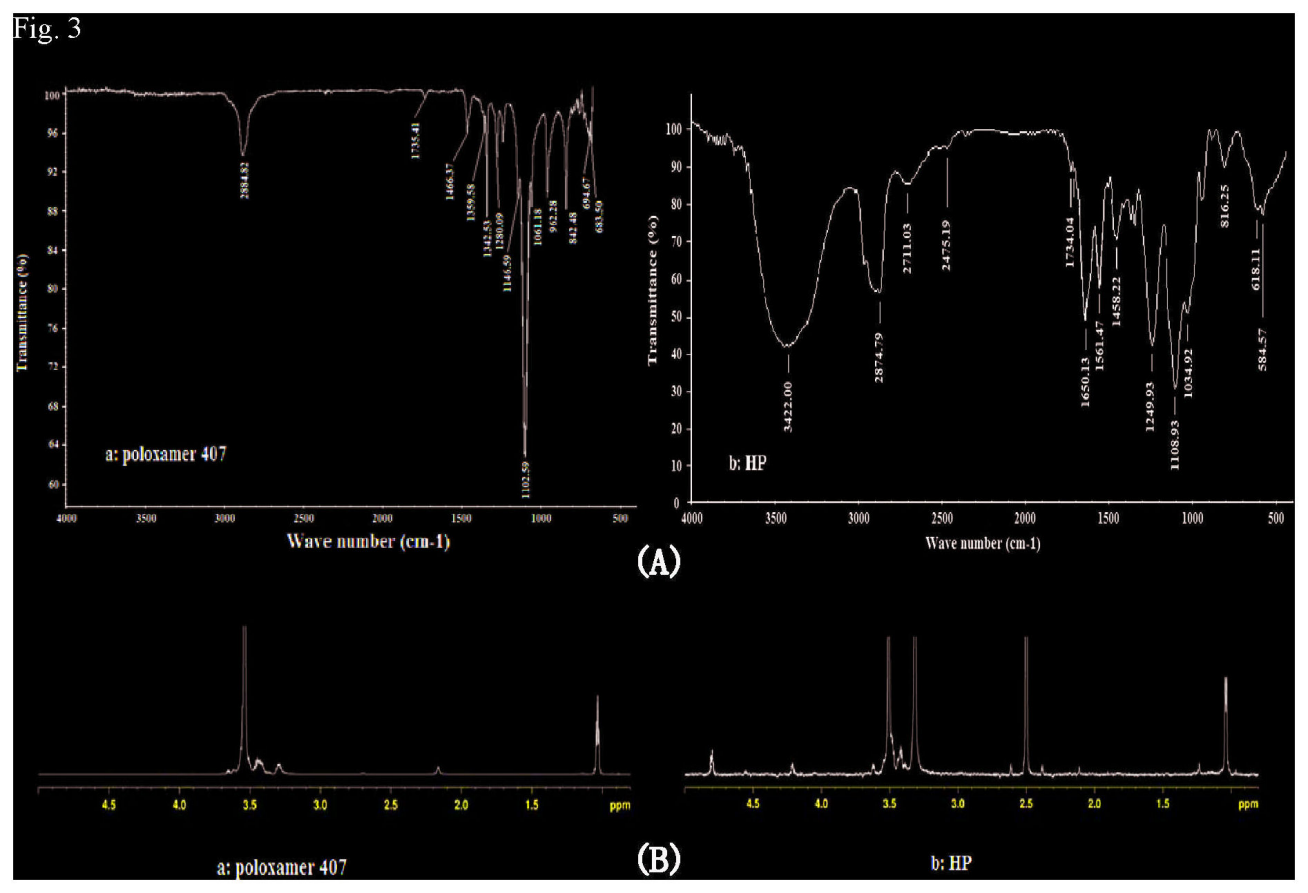
FTIR spectra of poloxamer and ^1^H-NMR spectra of Poloxamer 407 and HP. (A: FTIR spectra; B: ^1^H-NMR spectra. a: Poloxamer 407; b: HP).

HP copolymer was synthesized by conjugating MATP to heparin using EDC and NHS as coupling agents. The chemical structure of HP was determined using FTIR and ^1^H NMR. In the ^1^H NMR characterization, the typical peaks of heparin appeared at 1.2 ppm, 3.2 ppm, and 4.2 ppm [32,33]. In the FTIR characterization, the typical peaks of heparin appeared at 3300-3600 cm^−1^ and 1650 cm^−1^ [32,33]. As shown in FTIR spectra (Figure 3A), HP had the same carbonyl stretching vibration at about 1600 cm^−1^ as heparin. In addition, HP was characterized by a new absorption peak at 1736 cm^−1^, which belonged to carbonyl vibration of HP complex. A new band at 3300–3600 cm^−1^ was also observed in HP, which can be identified as hydroxyl groups of conjugated heparin. As shown in ^1^H-NMR spectra (Figure 3B), there were characteristic chemical shifts of PPO and PEO (d =1.14, 3.4, and 3.5 ppm for –CH_3_, –CH,–CH_2_ in group of PPO, d = 3.65 ppm for –CH_2_ of PEO). The rest of resonance signals indicated the presence of heparin in HP. From the spectral results, HP copolymer was successfully synthesized by EDC/NHS method. The amount of heparin associated with HP was 52.4%. The reduced amount of heparin in HP was most likely attributable to the changes in negative charge density and conformation, which were caused by the poloxamer conjugation.

As shown in Table 2, HP solution at different levels of HP concentration showed positive anti-coagulant activities in recalcification time test, compared with LMWH solution. Since the anti-coagulant activity of HP corresponded to the bioactivity of heparin, the synthesized HP exhibited reduced bioactivity of heparin about 30.6%.

**Table 2:** Recalcification time test.

*Group*	*Dose (mg/ml)*	*Recalcification time (min)*
Control		6.2±0.4
LMWH	0.5	45.2±1.6
HP (low level)	2.9	42.4±2.3
HP (medium level)	5.8	82.3±3.6
HP (high level)	9.5	139.6±6.8

### 2: Gelation temperature and rheological behavior

**Figure 4:**
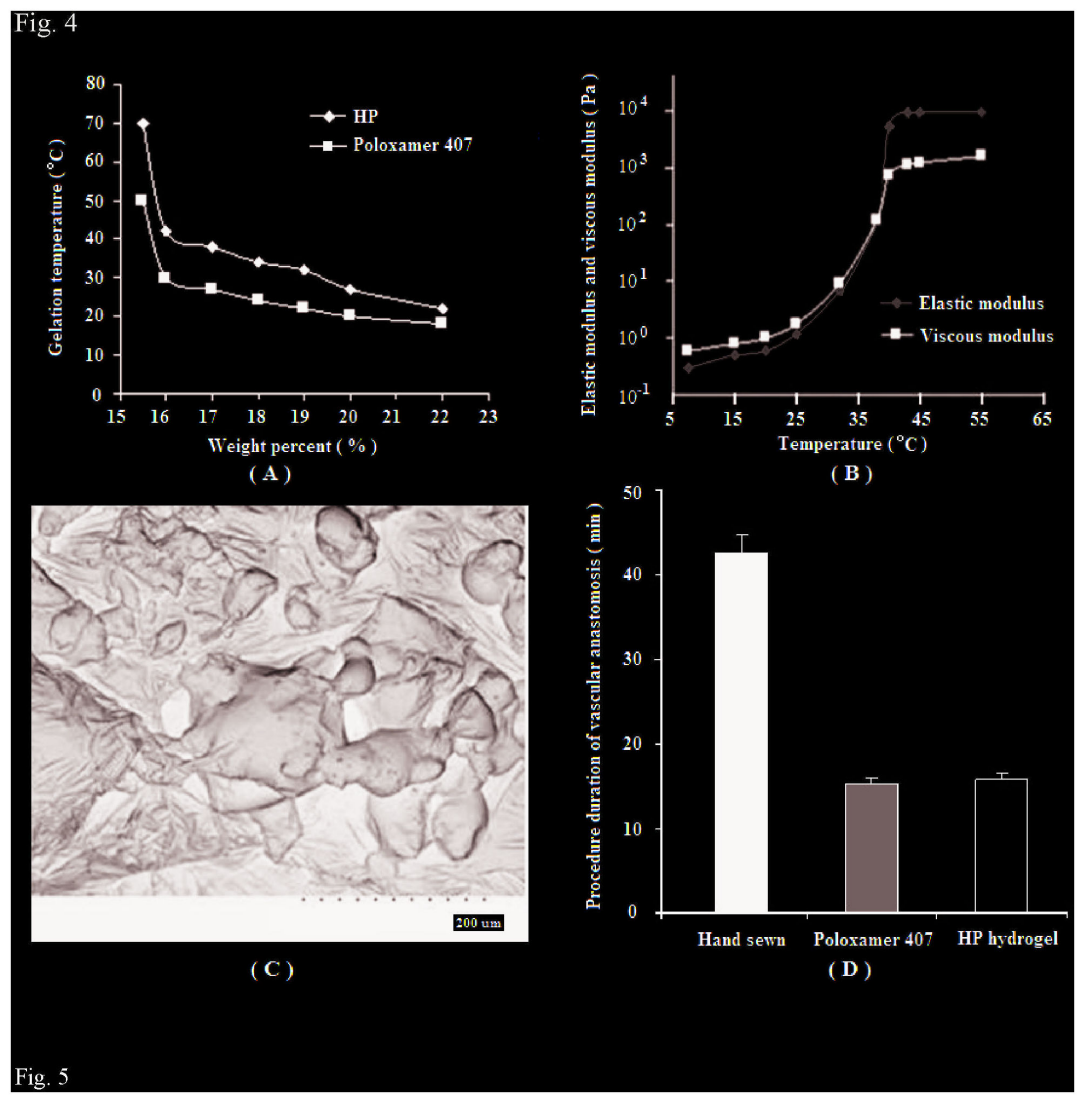
HP gelation temperature, viscous moduli and elastic moduli, SEM image and procedure duration of different vascular anastomosis. (A:Gelation temperature of poloxamer 407 and HP; B: Viscous moduli and elastic moduli of the 18wt% HP hydrogel in PBS; C: SEM images of the surface morphology of the lyophilized HP hydrogel at 18 wt%; D: Procedure duration of different vascular anastomosis).

The temperature-dependent sol-gel transition behaviors of poloxamer 407 and HP in PBS were shown in Figure 4A. Phase diagrams of these samples were determined by the vial-tilting method in a temperature range of 0–60°C.

As shown in Figure 4A, HP hydrogel resulted from 18 wt% HP solution had the suitable gelation temperature (about 38 °C) above human normal temperature, which would be convenient for vascular anastomosis. Therefore the 18 wt% HP hydrogel was recommended as the optimum HP hydrogel in following tests and vascular anastomosis.

An oscillatory rheological experiment was performed to evaluate the viscous moduli and elastic moduli of 18 wt% HP hydrogel, which reflected the potential for the injectable scaffold in vascular anastomosis.

In the sol phase under 25°C, the elastic moduli and viscous moduli of polymer solutions were extremely low. As the temperature increased, elastic moduli and viscous moduli increased and reached up to 10^3^–10^4^ Pa in the gel phase (Figure 4B). The elastic modulus of the hydrogel eventually dominated when it achieved a maximum elastic modulus of approximately 10,000 Pa. Though not as high as the artificial bone scaffold (the order of magnitudes in MPa) [34], these values of HP were adequate to perform the desired injectable scaffold function in vascular anastomosis.

In general, the addition of chains to an amphiphilic copolymer causes changes in sol-gel transition behavior of the conjugated chains [35,36]. From the results, the grafted heparin in the HP structure increased the critical gelation concentration (CGC) of poloxamer 407. As shown in Figure 4A and 4B, HP copolymer maintained the thermosensitive nature as well as injectable scaffold function, which would play important roles for the application of HP hydrogel in medical adhesive anastomosis.

### 3: Micromorphology of HP hydrogel

The micromorphology of HP hydrogels with different HP concentrations were observed by SEM and little differences were found.

As shown in Figure 4C, HP hydrogel (18 wt%) had a hollow structure similar as the porous sponge, in which the inner pores of the hydrogel were interconnected with irregular shape. The sponge-like structure of HP hydrogel may offer two benefits. First, it can serve as skeletal structure to provide strong physical support for the opening of vascular port during vascular anastomosis. Second, this structure may also increase the surface area to maximize the exposure of the grafted heparin on the HP. As a result, this novel HP material can efficiently benefit the operation process and prevent restenosis during and after vascular anastomosis.

### 4: Procedure duration of vascular anastomosis

The results were summarized in Figure 4D. The time taken to perform the anastomoses in the first group (hand-sewn anastomosis) varied from 40 to 45 minutes (Mean=42.3min), whereas the time taken to perform the anastomoses in the second group (anastomosis using poloxamer 407, Mean=15. 2 min) and in the third group (anastomosis using HP hydrogel, Mean=15.6min) were significantly shortened (*P* <0.05). Furthermore, there was little difference in the procedure duration of vascular anastomosis between the second group (anastomosis using poloxamer 407) and the third group (anastomosis using HP hydrogel) (*P*>0.05). The efficiency of poloxamer 407 as a thermosensitive filling agent in medical adhesive anastomosis has been reported in a previous study [1]. In this study, a formulation of 16.5 wt% P407 containing 0.25 wt% BSA was able to initiate phase transition at 30 °C and achieve a maximal elastic modulus of approximately 10,000 Pa at a temperature of 40 °C. The heated poloxamer easily stabilized an open lumen and allowed precise approximation of the intima. From the comparison, HP hydrogel showed similarly high efficiency as poloxamer 407 hydrogel in the procedure of medical adhesive anastomosis.

### 5: Imaging evaluation

**Figure 5:**
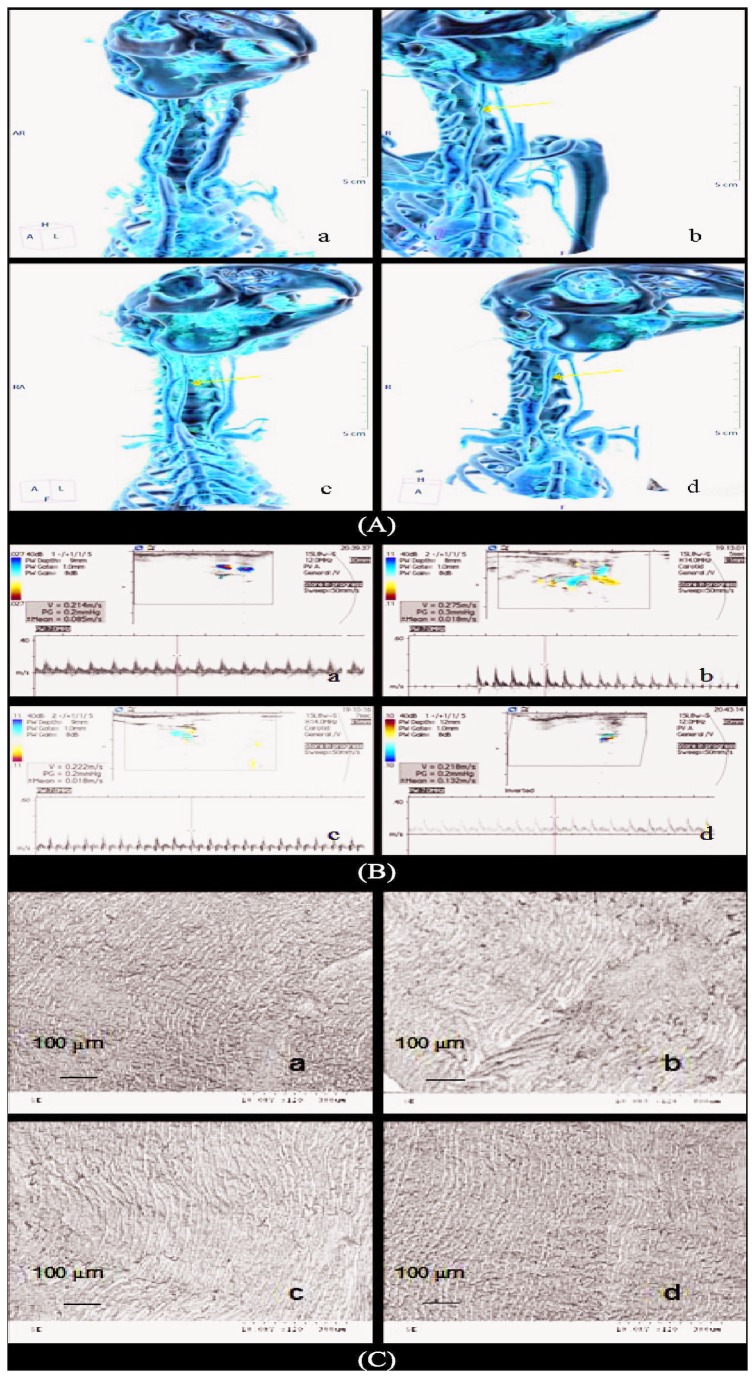
Postoperative CT angiograms, ultrasound Doppler images, and scanning electron microscopes of experimental groups. (A: Postoperative CT angiograms; B: Ultrasound Doppler images performed at 6 weeks after operation; C: Scanning electron microscope of different vascular anastomosis after 12 weeks. a: Sham-operated group, b: hand-sewn group, c: poloxamer 407 group, d: HP hydrogel group).

In order to evaluate the recanalization of blood flow at the jugular artery after vascular anastomosis, sixteen slice spiral CT and Ultrasound Doppler were used in this experiment. As shown in Figure 5A, postoperative CT angiograms performed at 6 weeks confirmed equivalent anastomoses using the poloxamer 407, HP hydrogel and hand-sewn techniques to sites of anastomoses. Little differences were found in the postoperative CT angiograms of the three groups (*P* > 0.05). Ultrasound Doppler studies also showed no observable differences in vessel diameter (2.1± 0.23 mm versus 2.2 ± 0.19 mm, *P* > 0.05) and confirmed patency of all anastomoses with similar volumetric flow rates (hand-sewn group: 0.225 ± 0.005 m/s, poloxamer 407 group: 0.236 ± 0.021 m/s, HP hydrogel group: 0.251 ± 0.324m/s, *P* > 0.05) (Figure 5B).

To investigate the prognosis and safety of HP hydrogel in vascular anastomosis, scanning electron microscopy (SEM) was performed at 12 weeks after vascular anastomosis operation. As shown in Figure 5C, persistent intimal damages to the luminal surface of the sutured anastomoses were observed in all the operation groups. From the images, HP hydrogel group showed the best recovery of the intima, followed with poloxamer 407 group and hand-sewn group.

### 6: Histological examination

**Figure 6:**
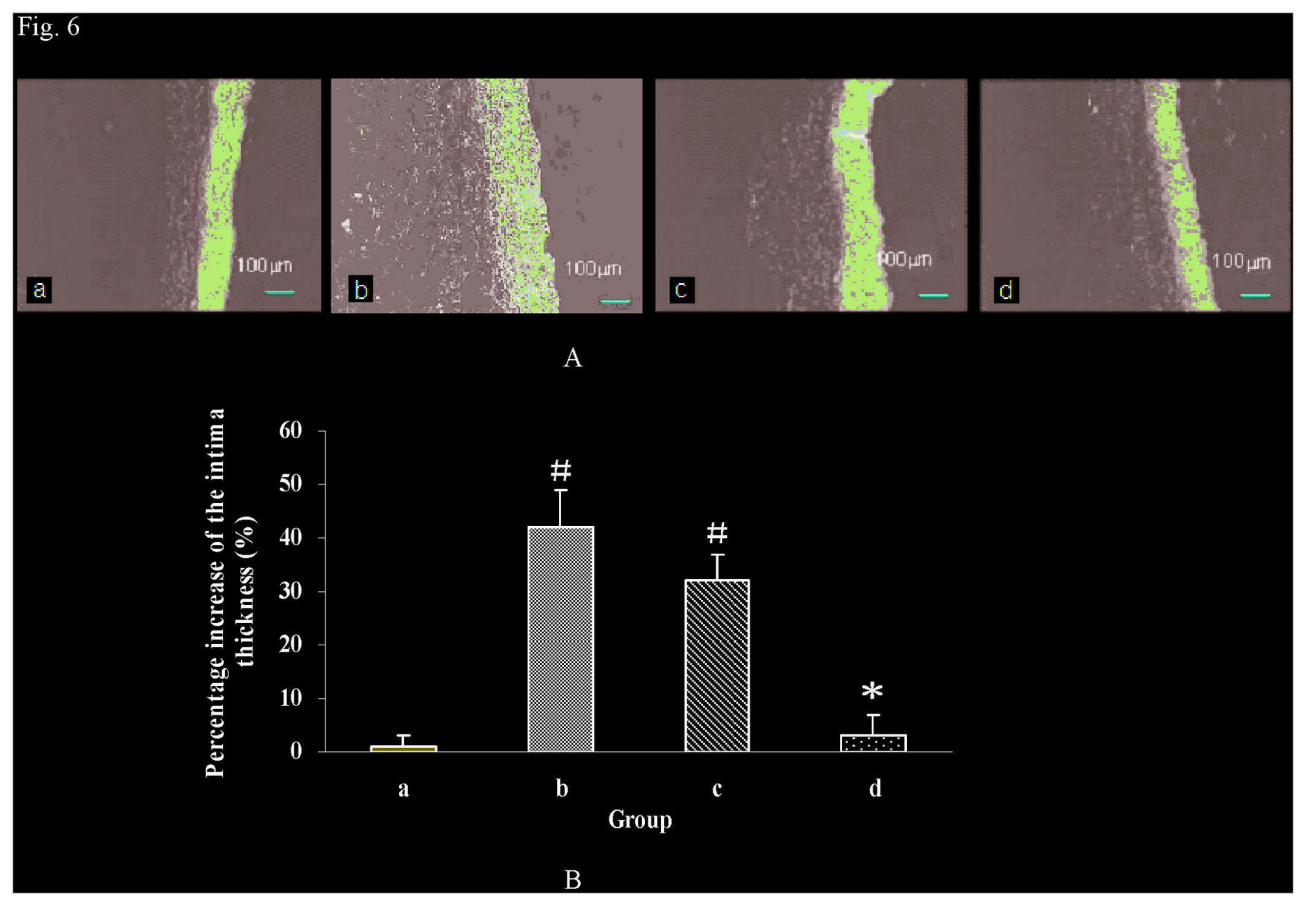
Histological observation and the percentage increase of the intima thickness at week 12 of experimental groups. (a: Sham-operated group, b: hand-sewn group, c: poloxamer 407 group, d: HP hydrogel group. Note: # *P*<0.05 versus the normal group; * *P*<0.05 versus the hand-sewn group and poloxamer 407 group. ).

The pathological changes of local vascular tissues and the new intima hyperplasia of experimental vascular segments were investigated by histological examination. The intima of the aorta was smooth with only one layer of endothelial cells in the control group. Among the experimental groups after anastomosis, the intima at the junction of broken arteries was damaged, which would activate platelet functions and resulted in the increased thickness of intima. Figure 6A presents the representative images. Comparing to the surgery-free control group, the intima thickness at the anastomosis junction in the hand-sewn group and poloxamer 407 group both significantly increased (*P*<0.05) (Figure 6B). However, there was no similar increase in the intima thickness in HP hydrogel group (*P*>0.05). In addition, little inflammation and fibrosis were observed in HP hydrogel group by histological examination.

## Conclusions

Low molecular weight heparin (LMWH) is known for its anti-fibrotic and anti-coagulant effects. Several laboratories have demonstrated that heparin can suppress the entry of smooth muscle cells (SMC) into a proliferative S-phase in response to some mitogens, but their mode of action remains unclear [37,38]. Heparin also inhibits experimental restenosis [39]. Poloxamer 407 is an attractive biopolymer for the preparation of thermosensitive hydrogels. Previous research has proved its potential as a thermosensitive filling agent in medical adhesive anastomosis [1].

In this paper, we attempt to combine the therapeutic advantages of LMWH and poloxamer for vascular anastomosis by synthesizing a novel thermosensitive HP copolymer and its hydrogel. In *in vitro* studies, HP copolymer displayed special gel-sol-gel phase transition behavior similar as poloxamer 407. With sponge-like structure, HP hydrogel (18wt%) had suitable elastic moduli in medical adhesive anastomosis. In our animal studies, we performed end-to-end anastomosis in the rabbit common jugular artery and compared the performance of HP-hydrogel to the poloxamer and hand-sewn controls. The results of Doppler ultrasound, CT angiograms, SEM and histological examination (Figure 5 A–C and Figure 6) all indicated that HP hydrogel treatment was more efficient than the standard hand-sewn operations for achieving vascular anastomosis. The findings also proved that HP hydroel was superior to poloxamer 407 hydrogel for restoring the functions and epithelial structure of the broken vessel junctions after operation.

Despite the exciting results in this paper, for this novel hydrogel to achieve the expected translational success, more in depth *in vivo* studies on issues such as the degradation mode of HP in blood circulation, the repeatablity under various conditions of anastomoses, and the stability of HP hydrogel will be needed. We are currently studying these issues in large animal models that are more clinically relevant. With further investigation, HP-based hydrogel can be developed as a clinically valuable choice to improve the quality and safety of vascular anastomosis.
