# Correction: Evaluation of a Novel Thermosensitive Heparin-Poloxamer Hydrogel for Improving Vascular Anastomosis Quality and Safety in a Rabbit Model

**DOI:** 10.1371/annotation/83ee5a05-d305-4a27-bf66-7e7dce37a85e

**Published:** 2013-09-05

**Authors:** Ying-Zheng Zhao, Hai-Feng Lv, Cui-Tao Lu, Li-Juan Chen, Min Lin, Ming Zhang, Xi Jiang, Xiao-Tong Shen, Rong-Rong Jin, Jun Cai, Xin-Qiao Tian, Ho Lun Wong

There was an error in the Funding statement. The correct version of the statement is available below.

This research was supported by the National Natural Science Funds (Grant No. 81272160, 81360195, 813715583 and 81301982), Zhejiang Provincial Natural Science Foundation (Grant No. LY12H30003 and LY12H18001), Zhejiang Provincial Foundation for Health Department (Grant No. 2011ZDA017, 2012KYB126 and 2012KYA134), Zhejiang Provincial Project of Key Scientific Group (Grant No. 2010R50042 and 2012R10042-06), Zhejiang Educational Fund (Grant No. Y201017307). The funders had no role in study design, data collection and analysis, decision to publish, or preparation of the manuscript. 

