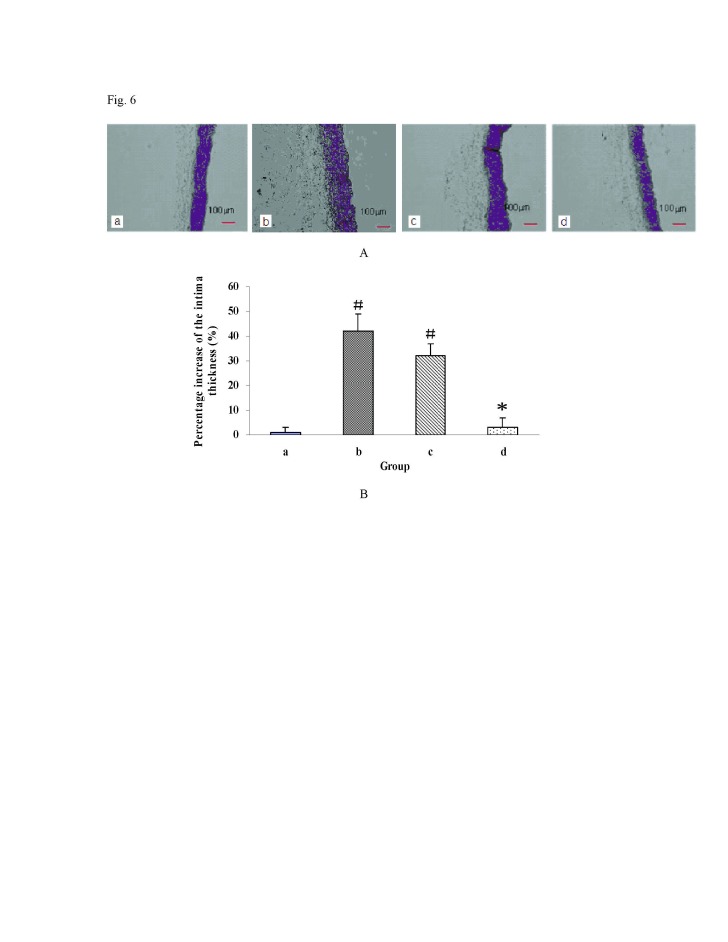# Correction: Evaluation of a Novel Thermosensitive Heparin-Poloxamer Hydrogel for Improving Vascular Anastomosis Quality and Safety in a Rabbit Model

**DOI:** 10.1371/annotation/9ba3d2e7-10c6-4e5c-96a8-ac331b4c2a83

**Published:** 2013-10-10

**Authors:** Ying-Zheng Zhao, Hai-Feng Lv, Cui-Tao Lu, Li-Juan Chen, Min Lin, Ming Zhang, Xi Jiang, Xiao-Tong Shen, Rong-Rong Jin, Jun Cai, Xin-Qiao Tian, Ho Lun Wong

Due to errors in the production process, Figures in the article were presented incorrectly. Accurate versions of the Figures are available below.

Figure 1: 

**Figure:**
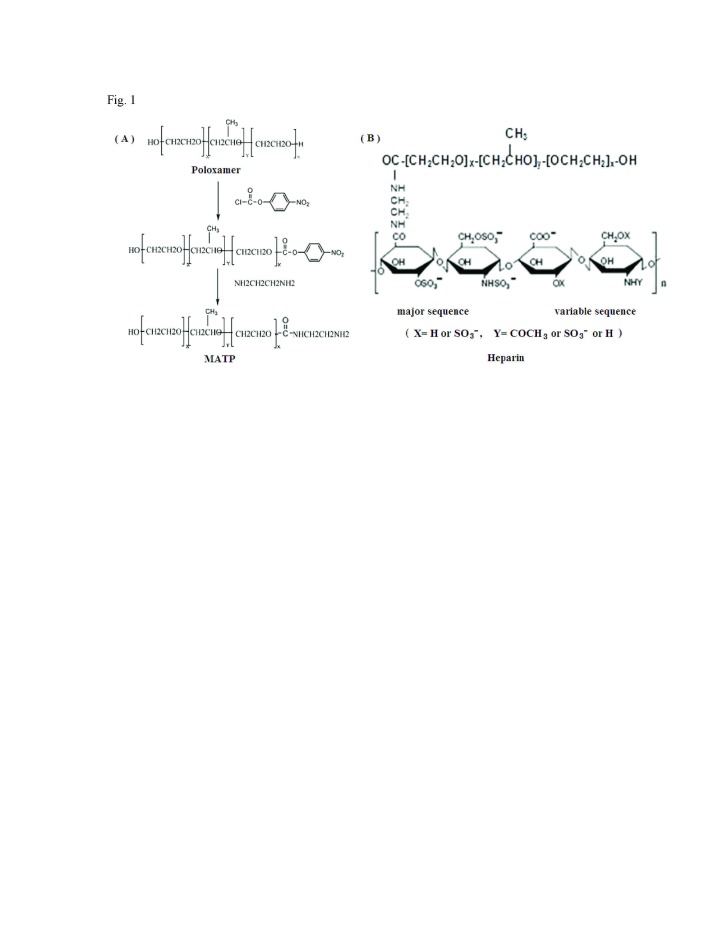


Figure 2: 

**Figure:**
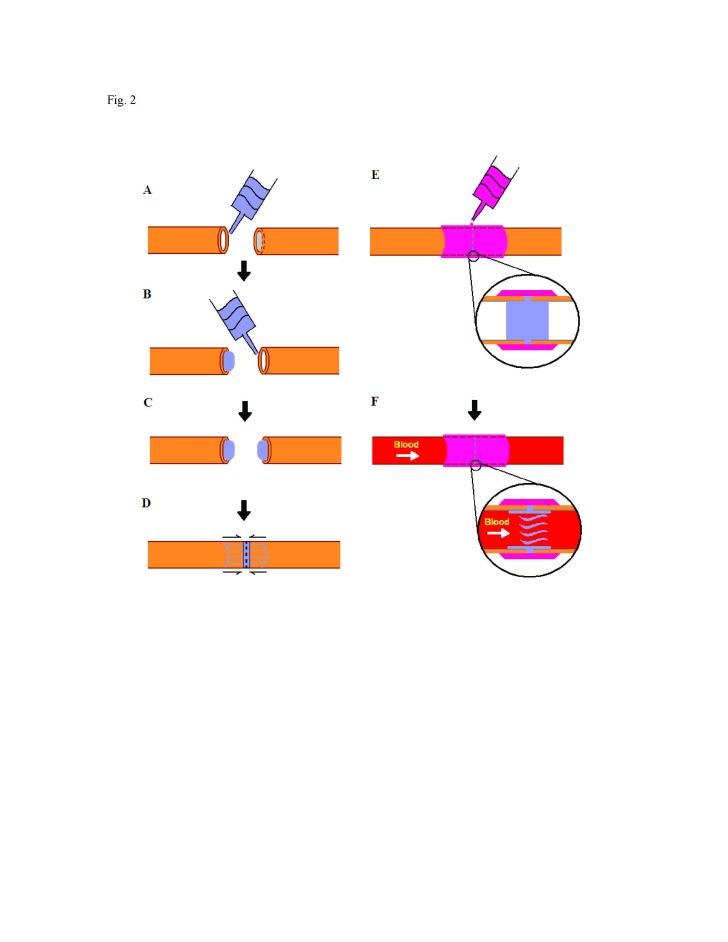


Figure 3: 

**Figure:**
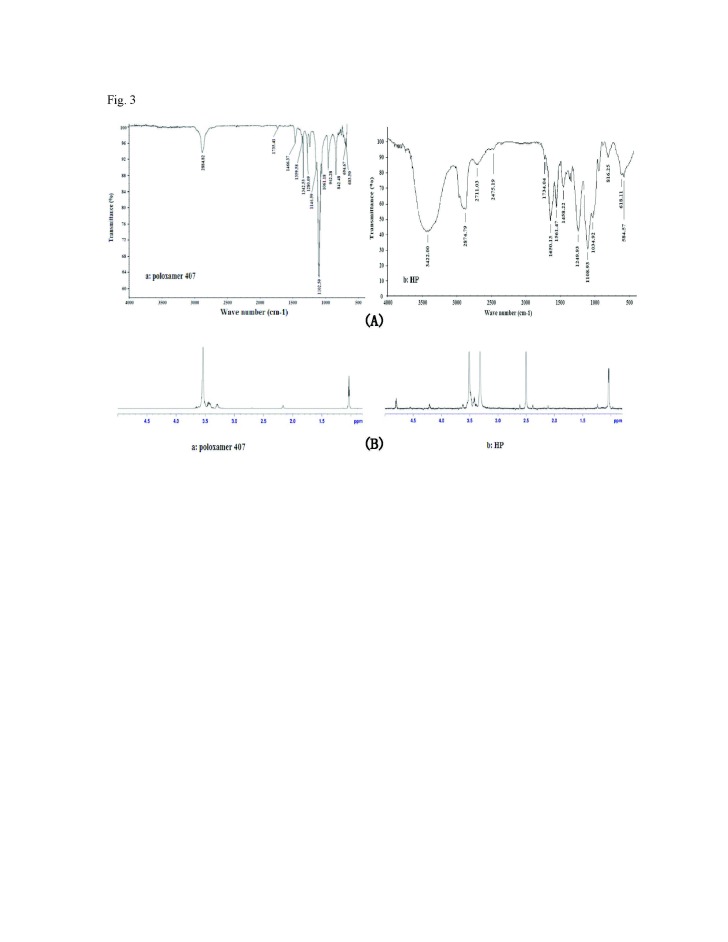


Figure 4: 

**Figure:**
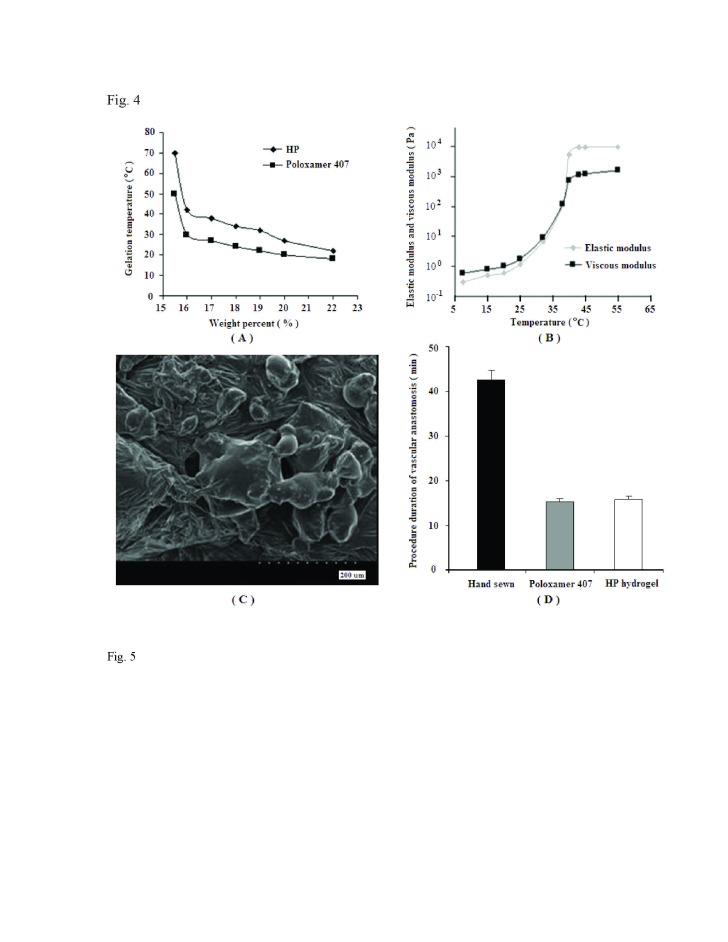


Figure 5: 

**Figure:**
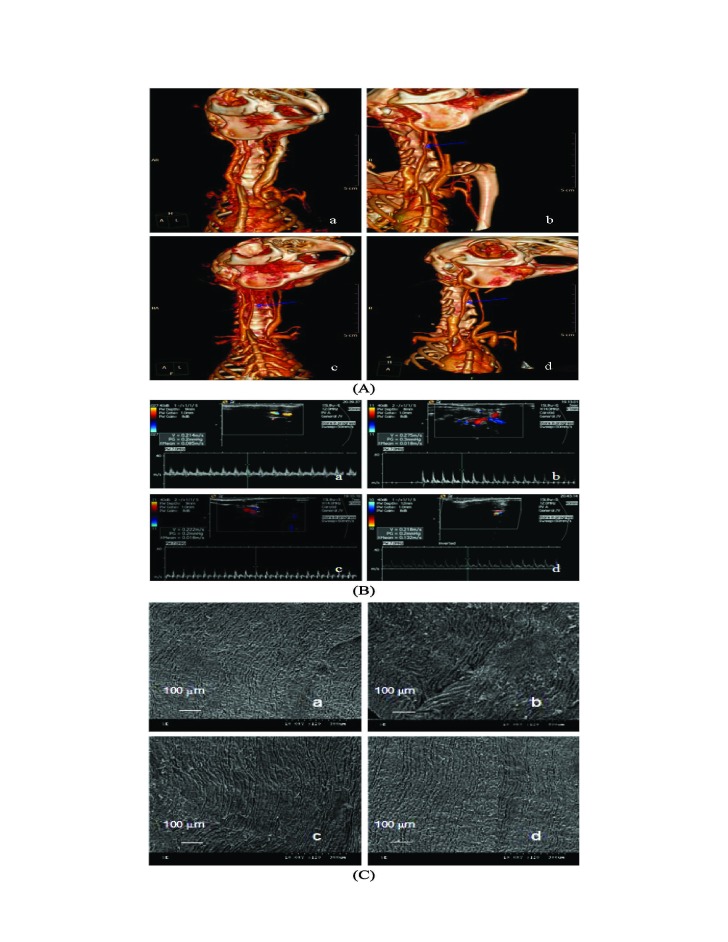


Figure 6: 

**Figure:**